# Typing methods based on whole genome sequencing data

**DOI:** 10.1186/s42522-020-0010-1

**Published:** 2020-02-18

**Authors:** Laura Uelze, Josephine Grützke, Maria Borowiak, Jens Andre Hammerl, Katharina Juraschek, Carlus Deneke, Simon H. Tausch, Burkhard Malorny

**Affiliations:** Department for Biological Safety, German Federal Institute for Risk Assessment, BfR, Max-Dohrn Straße 8-10, 10589 Berlin, Germany

**Keywords:** Whole genome sequencing, Typing, Methods, Comparison, Bioinformatics tools

## Abstract

Whole genome sequencing (WGS) of foodborne pathogens has become an effective method for investigating the information contained in the genome sequence of bacterial pathogens. In addition, its highly discriminative power enables the comparison of genetic relatedness between bacteria even on a sub-species level. For this reason, WGS is being implemented worldwide and across sectors (human, veterinary, food, and environment) for the investigation of disease outbreaks, source attribution, and improved risk characterization models. In order to extract relevant information from the large quantity and complex data produced by WGS, a host of bioinformatics tools has been developed, allowing users to analyze and interpret sequencing data, starting from simple gene-searches to complex phylogenetic studies. Depending on the research question, the complexity of the dataset and their bioinformatics skill set, users can choose between a great variety of tools for the analysis of WGS data. In this review, we describe the relevant approaches for phylogenomic studies for outbreak studies and give an overview of selected tools for the characterization of foodborne pathogens based on WGS data. Despite the efforts of the last years, harmonization and standardization of typing tools are still urgently needed to allow for an easy comparison of data between laboratories, moving towards a one health worldwide surveillance system for foodborne pathogens.

## Historical perspective on typing methods for foodborne pathogens

Following the establishment of the germ theory of disease, postulated by Louis Pasteur in the late 1850s, and extended by Robert Koch in the 1880s, major advances in isolation and cultivation techniques of bacterial organism were made, making it possible for microbiologists to clearly differentiate bacteria from each other, even within a species, thus pushing the development of prokaryote taxonomy [1]. Initially, physiological, biochemical and other phenotypic properties served as markers for species identification. In the 1930s, serotyping was one of the first approaches to differentiate bacteria based on antigen-antibody reactions on a species and subspecies level. Later, in the 1950s, phage typing schemes e.g. for *Staphylococcus* spp., were developed to be even more discriminative [2]. From the beginning, these schemes were used to trace the source of infections.

The discovery of nucleic acids, the postulation that genetic information is embedded in the DNA, and the description of the structure of the DNA molecule by Watson and Crick in the middle of the 1950s, formed the foundation of the new field of Molecular Biology [3]. At the beginning of the 1980s, Tenover and colleagues [4] developed the first bacterial strain typing method based on nucleic acids as marker molecules. It followed the discovery that the number and sizes of plasmids within different bacterial strains vary considerably, and that therefore it is possible to use plasmids naturally occurring in many genomes, to distinguish strains in an outbreak investigation. Although the first DNA sequencing method (which made it possible to determine the exact base pair sequence of a DNA fragment) was developed by Maxam-Gilbert and Sanger as early as 1977, it did not initially find broad application in microbial typing. Instead, pulsed-field gel electrophoresis, developed in the late 1980s, became the universal and widely used gold standard method for bacterial strain typing for the following two decades [5]. During pulsed-field gel electrophoresis, genomic DNA is fragmented with rare-cutting enzymes and the resulting size and number of DNA fragments form a stable and reproducible restriction pattern, which can be compared between different strains. DNA sequencing remained a specialized and expensive method until the late 1980s, when the polymerase chain reaction was developed by Kary Mullis and Michael Smith [6]. Using this method, a specific piece of DNA can be exponentially amplified, before it is separated by size in an electric field and visualized by intercalating dyes. The polymerase chain reaction transformed the sequencing process, significantly improving the applicability of Sanger-sequencing in diagnostics. Since then, many sequence-based typing approaches for the detection and typing of foodborne pathogens have been developed. One of the most successful sequence-based typing approaches is the concept of multilocus sequence typing (MLST), initially proposed for the pathogen *Neisseria meningitidis* in 1998 [7, 8]. Since then numerous MLST schemes were developed and are currently applied for hundreds of pathogens (http://pubmlst.org). In general, MLST typing involves the amplification of seven loci of housekeeping gene by PCR, followed by DNA sequencing of the resulting PCR fragments. Specific DNA sequences are then matched to allelic profiles. A single nucleotide variation at any of these loci defines a different allele and informs the sequence type (ST). MLST detects changes at DNA level that cannot be inferred from the phenotype, such as serotyping or multilocus enzyme electrophoresis (MLEE). Multilocus sequencing generates comparably small data files, which contain non-ambiguous information and which can be easily shared with other laboratories. Generally, the discriminatory power of MLST is comparable or slightly better than traditional serotyping [9]. Nevertheless, 7-gene MLST is often not discriminative enough to be useful for outbreak detection. Because of this, the PCR-based typing method multilocus variable-number tandem-repeat analysis (MLVA) was developed to discriminate between highly related strains [10]. This approach is based on the detection of repetitive tandem DNA units within various loci. Repeating units occur of approximately 1–100 base pairs in length. The number of tandem repeats can change by slipped strand mispairing mechanism with each generation, making it possible to infer relatedness of bacteria from the variation in the tandem repeat units. Because MLVA has been proven in outbreak studies as a fast tracing tool with increased resolution compared to pulsed field gel electrophoresis (PFGE), the method has been standardized for certain pathogenic subtypes [11, 12].

The advance of WGS has provided new opportunities to investigate the evolution of foodborne pathogens even over short time periods [13, 14]. WGS provides unprecedented resolution in discriminating highly related strains. Although PFGE and MLVA were milestones in bacterial strain typing, they were not informative enough for certain types of analysis, such as evolutionary studies and spatiotemporal investigations. In contrast, WGS offers ultimate resolution for surveillance and outbreak investigations, source attribution, genomic studies, as well as genomic information for the prediction of phenotypes (serotyping, antimicrobial resistance, biofilm formation, pathogenicity and virulence). Many approaches and bioinformatics tools have been developed to analyse and extract the relevant genomic data. Here, we summarize the most important and recent concepts for typing foodborne pathogens.

## Phylogenomic analyses of foodborne pathogens

One of the great benefits of WGS lies in comparative genomics, which allows the inference of the phylogenetic relationship between a set of bacterial strains. This provides valuable information for the tracking of the outbreak source and for the identification of clonal strains.

In a first step, the similarity between different genomes is estimated by different approaches further described in Table 1. Subsequently this is followed by a clustering step to infer phylogenetic relationships and clusters. Two methods, gene-by-gene (also known as multi-locus sequence typing) and Single-Nucleotide Polymorphism (SNP)-based approaches are commonly distinguished. Both approaches have in common that a distance matrix between a set of strains can be derived (see below for details), which allows the construction of a phylogenetic tree via various clustering techniques (e.g. neighbor-joining trees, minimum-spanning trees, hierarchical clustering). Either approaches can be used to define cluster types and cluster addresses: all samples within a specified distance threshold belong to the same cluster type. A cluster address e.g. SNP address [15], or Hierarchical Clustering of core genome MLST (cgMLST) sequence types (HierCC) [16] is the combination of cluster types with a set of different distance thresholds. It provides a quick interpretation of the degree of similarity of a set of samples related to an outbreak, super-lineage or eBurst group.

**Table 1:** Phylogenetic approaches

Method	Approach	Reference	Primary result	Secondary result
cgMLST	Alignment to scheme of core genes	Set of allele sequences for set of core genes	Allele distance matrix	Minimum-spanning tree
wgMLST	Alignment to scheme of core and accessory genes	Set of allele sequences for set of core and accessory genes	Allele distance matrix	Minimum-spanning tree
SNP	Mapping to reference	Closely related reference genome	Core SNP alignment, SNP distance matrix	Neighbor-joining tree
				Maximum- likelihood tree
split K-mer based SNP detection	Pairwise K-mer comparison	No reference	Core SNP alignment, SNP distance matrix	Neighbor-joining tree
MinHash	Pairwise MinHash comparison and clustering	No reference	MinHash distances, clustering information	Neighbor-joining tree

### cgMLST

To analyse the genetic similarity between genomes in a species the initial 7-gene multi-locus sequence typing approach has been upscaled to hundreds or thousands of gene loci [8, 17]. Core genome MLST (cgMLST) is a gene-by-gene approach which compares genomes using a large number of gene loci. In practice, genome assembly data is aligned to a scheme – a set of loci and a collection of associated allele sequences. The allele calling step yields either the allele number of an allele sequence already present in a scheme or assigns a new allele number. As a result of cgMLST allele calling, each isolate is characterized by its allele profile, i.e. the set of allele numbers for each locus. The sum of differently assigned allele numbers between a pair of samples determines the allele difference (either accounting for missing loci or the absolute difference) and the cross-comparison of a set of samples yields the allele distance matrix.

Finally, cgMLST analyses can be turned into a phylogeny via different strategies, e.g. single-linkage hierarchical clustering, neighbor-joining (NJ) or minimum spanning (MS) trees [18]. The choice of method depends on the ancestral divergence (high divergence is better reflected in NJ trees), computational considerations (MS trees is less demanding) and presence of missing data.

### cgMLST schemes

**Fig. 1:**
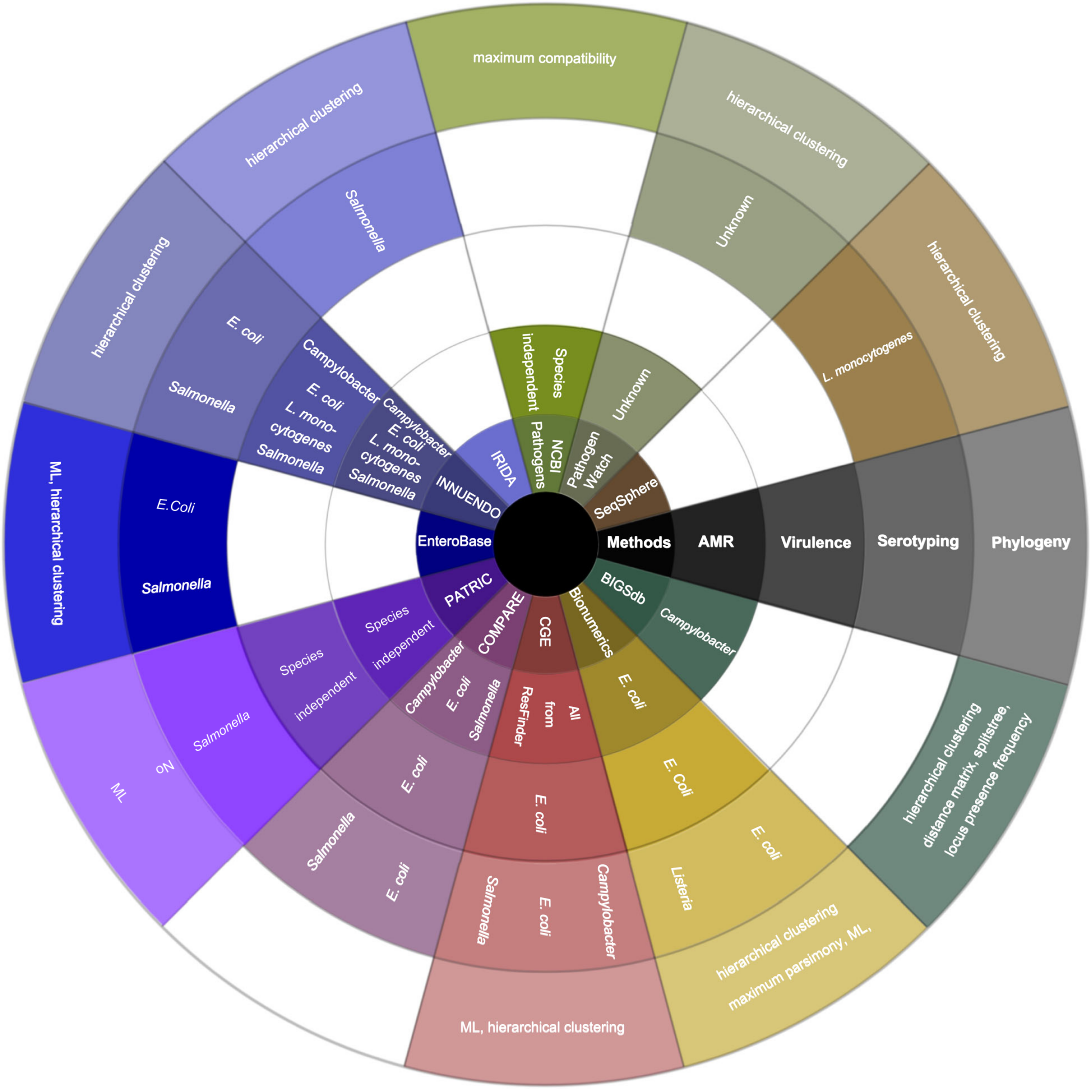
Wheel of tools and supported methods. Provided methods: Antimicrobial resistance gene detection (AMR), Virulence factor search (Virulence), Serotyping and Phylogeny (highlighted in black/grey) by selected tools (BIGSdb, Bionumerics, CGE, COMPARE, PATRIC, EnteroBase, INNUENDO, IRIDA, NCBI Pathogens, PathogenWatch and SeqSphere). Organisms for which a methodology is supported by a tool are specified. For phylogeny, the underlying methods are mentioned. White fields indicate that functionality is not supported by the respective platform. ML = Maximum Likelihood

Central to the cgMLST approach is the definition of a cgMLST scheme [17]. A given scheme consists of a defined set of loci and a collection of alleles for each locus which are typically numbered (allele numbers). A scheme is created by collecting a large number of genomes of a species and identifying the set of loci present in the majority (frequently > 95%) of the genomes of a taxonomic grouping [19, 20]. Schemes exist for various species (Table 2). In some cases (e.g. *Listeria monocytogenes*) various schemes exist for the same species. Although they may lead to similar conclusions [21], and are likely to yield phylogenetic trees with overall similar topology, cgMLST sequence types derived from different schemes are not directly comparable as they may contain different loci, loci names, or other loci orders, etc. Even schemes with the exact same locus definitions, but hosted on different services (e.g. Enterobase and Ridom SeqShere+, compare Fig. 1) are not comparable since the allocation of novel allele numbers are not synchronized and the same allele number relates to different allele sequences.

**Table 2:** Available cgMLST schemes

Provider	Website	Publically accessible	Species
Enterobase	http://enterobase.warwick.ac.uk/	Yes	*Salmonella, Escherichia/Shigella, Clostridioides, Vibrio, Yersinia, Helicobacter, Moraxella*
Pasteur Institute	https://bigsdb.pasteur.fr/	Yes	*Klebsiella pneumoniae***/** *quasipneumoniae*/*variicola*, *Listeria, Bordetella, Corynebacterium diphtheriae, Yersinia, Leptospira Elizabethkingia anopheles*/*meningoseptica*/*miricola*
Ridom	https://cgMLST.org/ncs	Yes	*Acinetobacter baumannii, Brucella melitensis, Clostridioides difficile, Enterococcus faecalis, Enterococcus faecium, Escherichia coli, Francisella tularensis, Klebsiella pneumoniae/variicola/quasipneumoniae, Legionella pneumophila, Listeria monocytogenes, Mycobacterium tuberculosis/bovis/africanum/canettii, Mycoplasma gallisepticum, Staphylococcus aureus*
Applied Maths	http://www.applied-maths.com/applications/wgmlst	No	*Acinetobacter baumannii, Bacillus cereus, Bacillus subtilis, Burkholderia cepacia complex, Brucella* spp*.*
			*Campylobacter coli - C. jejuni, Citrobacter* spp.*, Clostridium difficile, Cronobacter* spp.*, Enterobacter cloacae, Enterococcus faecalis, Enterococcus faecium, Enterococcus raffinosus, Escherichia coli / Shigella, Francisella tularensis, Klebsiella aerogenes, Klebsiella oxytoca, Klebsiella pneumoniae, Legionella pneumophila, Listeria monocytogenes, Micrococcus* spp*., Mycobacterium bovis, Mycobacterium leprae, Mycobacterium tuberculosis, Neisseria gonorrhoeae, Pseudomonas aeruginosa, Salmonella enterica, Serratia marcescens, Staphylococcus aureus, Staphylococcus epidermidis, Staphylococcus pseudointermedius, Streptococcus pyogenes*
INNUENDO/ chewBBACA	http://chewbbaca.online/	Yes	*Acinetobacter calcoaceticus/baumannii complex, Legionella pneumophila, Streptococcus pyogenes, Escherichia coli, Yersinia enterocolitica, Campylobacter jejuni, Salmonella*

### cgMLST vs wgMLST

Whole-genome MLST (wgMLST) can be viewed as an extension to cgMLST which uses – in addition to a set of core genome loci – also a set of accessory loci [20, 22, 23].

In principle, wgMLST can provide a higher resolution for closely linked clusters as the distance matrix is computed on a larger set of loci. Nevertheless a number of studies demonstrate that results derived from wgMLST and cgMLST approaches are often quite similar. For example, Pearce et al. [24] were able to demonstrate that there was no statistically significant difference in the discriminatory ability of cgMLST and wgMLST within a *S. enterica* serovar Enteritidis outbreak. This was further confirmed in a study analysing 145 *S. enterica* serovar Heidelberg strains involved in four distinct outbreak events [25]. Another study analyzing a diverse set of ~ 200 *Listeria monocytogenes* strain found that when comparing phylogenetic trees derived from wgMLST and cgMLST their topology were highly similar [26]. For the practical application, one can envision a first cgMLST analysis on a diverse dataset of a species followed by wgMLST for closely related (according to the cgMLST results) strains.

Since cgMLST is a stable typing method for bacteria within a species with many publically available schemes it facilitates global foodborne outbreak investigation [19, 20]. However, to date no worldwide agreed centrally organized allele nomenclature system exists. Assignment of allele numbers to novel alleles is currently done on local or systems with centrally curated nomenclature such as the Enterobase service and others (see section below). Although schemes can be shared, the sharing of analyses between different sites is impeded by the possibility to efficiently synchronize novel alleles. Furthermore, cgMLST results depend on the detailed trimming, assembly and alignment strategy. In our experience, different approaches can cause several allele differences (unpublished data).

### SNP calling and choice of reference

Another approach is the identification of single nucleotide polymorphisms (SNPs) that vary among strains. SNPs are detected by mapping sequence reads against a closely related reference genome and recording nucleotide differences [27]. For a set of strains, only reference positions that are covered by all query genomes are considered, which form a set of core SNPs. All possible combinations of pairwise SNP distances determine the SNP distance matrix which allows fast and simple phylogenetic analysis such as neighbor-joining trees. Moreover, the aligned core SNPs form the basis for a more detailed evolutionary analysis – typically maximum likelihood phylogenetic trees [28]. SNP-based analyses have been successfully applied in resolving large national and international outbreaks [27, 29, 30]. The choice of a reference is crucial for reliable SNP analyses [31]. Firstly, a high-quality, closed reference genome permits calling SNP positions with higher accuracy than a non-curated draft genome containing many contigs. Secondly, the reference is ideally closely related to the set of strains under investigation. If it is too distant, less reference positions will be covered and subsequently less SNPs discovered. Likewise if the set of query genomes contains one or more remotely linked isolates, the set of core SNPs will be reduced. Strategies for obtaining a good reference consist in choosing a genome from the same serogroup, 7-gene MLST or MLST clonal complex [15]. Other approaches estimate the average distance of the query genomes to a large set of potential reference genomes (https://gitlab.com/s.fuchs/refRank). Apart from the choice of reference, a number of algorithms and parameters need to be defined for calling, quality assuring and filtering SNPs [27, 32]. This can potentially hinder standardization within and between laboratories [33, 34].

There is a variety of tools available for SNP calling, such as SAMtools [35], GATK [36] and Freebayes [37]. Furthermore there are specialized pipelines for SNP calling from bacterial genomes, for example Snippy (https://github.com/tseemann/snippy), CFSAN SNP Pipeline [38], NASP [32] and BactSNP [39]. Other solutions are targeted to routine sequencing and SNP calling such as SnapperDB [15], which is essentially a database that stores variant call files from each isolate. This has the advantage that new strains can be compared to the database and a pairwise distance matrix can be updated quickly, which allows easy clustering and searching.

### Comparison of SNP and cgMLST

It has been shown that SNP and cgMLST (and wgMLST) analyses are congruent and both approaches are well suited and commonly applied for food outbreak analyses [24]. The cgMLST approach has the advantage that it uses a consistent set of conserved loci and allele definitions for an entire taxonomic group such as a species. Conversely, an allele difference between two strains may be explained by one or several mutations, thus indicating the intrinsically higher discriminatory power of SNP analyses. In particular, SNP results allow the application of detailed evolutionary models for true phylogenetic inference, based on the core SNP alignment. In practice, SNP analyses may be applied after defining a potential phylogenetic cluster after pre-clustering with e.g. cgMLST.

### K-mer based approaches

Apart from the commonly applied approaches discussed here, a number of novel approaches attempt to overcome the need of an a priori reference and scheme definition. K-mer based tools split WGS data into nucleotide blocks of a defined length *k*. The pair-wise comparison of the k-mer content between a set of genomes are useful to evaluate their phylogenetic relatedness. K-mer approaches are often applied in order to investigate the taxonomy of microorganisms [40] but are also used for sub-clustering, e.g. serovar prediction, antimicrobial resistance typing or mobile genetic elements identification (see sections below).

An interesting open-source tool is kSNP3 [41], which can detect SNPs between strains without the need of a reference genome. To do so it uses a k-mer based approach that can detect core SNPs between a set of strains and which can return parsimony, neighbor-joining and maximum-likelihood trees. kSNP3 was successfully applied for a retro-perspective outbreak detection [42, 43]. Another k-mer based approach, PopPUNK (Population Partitioning Using Nucleotide K-mers), exploits the estimated overlap of core and accessory genome between a pair of sequences using the MinHash algorithm [44, 45]. Based on this set of distance pairs, clusters are created using model fitting, either using a two-dimensional Gaussian mixture model or density-based hierarchical clustering (HDBSCAN). PopPUNK was shown to be able to successfully resolve diverse bacterial populations into strains (and detect similar clonal complexes as cgMLST). Another advantage of PopPUNK is that new genomes can easily be associated to existing clusters without the need to refit the model or recalculate all pairwise distances. Another novel tool for the analysis of highly similar sequences, such as those encountered in outbreak investigations is Split Kmer Analysis (SKA) [46]. This method detects split k-mers (pairs of k-mers which are separated by a single base) and employs those as markers for variation between closely-related genomes sequences. SKA has the advantage of being very rapid and memory-efficient and preliminary results show its use in identifying clusters in a retrospective epidemiology study [47].

### Phylogenetic tools

Given a core alignment resulting from a SNP analysis, a number of tools exist for subsequent phylogenetic analysis. Some fast and simple tools, such as fasttree, are able to estimate approximate maximum likelihood trees, however these may have limited accuracy [48]. A maximum likelihood based tool providing a large number of evolutionary models and bootstrap settings is RAxML (Randomized Axelerated Maximum Likelihood) [49]. Similarly, IQ-TREE is a fast and effective stochastic algorithm to infer phylogenetic trees by maximum likelihood [50]. The Bayesian method MrBayes infers phylogeny using a Markov chain Monte Carlo method [51]. BEAST is a similar program based on Bayesian analysis with a focus on time-scaled trees [52]. Although the Bayesian inference of phylogenies is computational expensive, it provides a large number of options and yields very accurate phylogenies. A recent evaluation shows that RaxML, as well as IQ-TREE, produce reasonably accurate trees in acceptable computational time [53]. Another tool, Gubbins, allows the phylogenetic inference of recombinant bacterial species (such as *Campylobacter* spp.), while mitigating the effect of horizontal sequence transfer on phylogenetic reconstructions [54]. To do so, it identifies regions containing elevated densities of base substitutions and constructs the phylogeny from the sequence outside of these regions.

## Pathotyping of foodborne pathogens using WGS data

The estimation of the pathogenic potential of a strain is based on the detection of associated virulence factors (VFs). These factors can be differentiated in six categories: i) adherence and colonization factors, ii) Type I to VI secretion systems, iii) immune evasion factors, iv) toxins, v) siderophores for iron absorption and vi) invasion genes [55]. WGS not only allows the detection of known VFs, but also makes it possible to identify new genes or gene variants that confer virulence to bacteria. The relatively high number of hypothetical proteins with unknown function, resulting from microbial genome annotation, implies the presence of further virulence factors within this ‘biological dark matter’. Virulence prediction can be difficult and often needs to be considered contextually, as illustrated by the fact that classical VFs can also sometimes be identified in non-pathogenic strains [56, 57]. The simple detection of the presence or the absence of VFs might therefore not be sufficient due to complex regulative pathways and the impact of mutations in regulators, which can cause an altered virulence as shown for *Streptococcus* spp. [58] and for *Staphylococcus aureus* where more surface proteins are expressed in the virulent strain [59]. Also, the loss of regulation genes, as it is the case for *Rickettsia prowazekii* that causes epidemic typhus in humans, leads to an increased pathogenicity [60]. Nevertheless, the detection of VFs is a relevant indication for the pathogenicity of most bacteria. Several computational approaches were developed to predict VFs by similarity to known virulence associated patterns. These methods can be differentiated into homology based search, detection of divergent sequence patterns or motifs and machine learning approaches.

One of the major ways to identify virulence genes in WGS data is the search for homologs to genes or proteins already known to be VFs. BLAST [61, 62] is one of the most flexible tools for this task and can be applied on sequencing reads, assembled genomes or protein level. Further, open-source tools running via command-line on nucleotide level include ABRicate (https://github.com/tseemann/abricate) and AMRFinderPlus [63] that require assembled genomes, Short Read Sequence Typing (SRST2) [64] for short read sequences as input and ARIBA that produces local assemblies after read mapping to reference genes [65]. The web-based VirulenceFinder (https://cge.cbs.dtu.dk/services/VirulenceFinder/) is an alternative for selected organisms such as *Escherichia coli* and *Staphylococcus* spp. with its own curated database that can also be downloaded and used in combination with open-source tools. There are several databases available that collect virulence associated genes as well as associated relevant information. Currently, the Virulence Factor Database (VFDB) [66] contains 1080 virulence factors of which 575 genes are experimentally verified and 3224 curated virulence factor related genes from 74 bacteria genera. While VFDB is restricted to bacteria, Victors, a manually curated database contains more than 5000 VFs from about 200 pathogens including bacterial, viral parasitic and fungal VFs, which also provides a customized online BLAST against its own database [67]. The Pathosystems Resource Integration Center (PATRIC) contains manually curated VFs and integrates VFs from both the VFDB and Victors for its data annotation and analysis service [68]. One major drawback of the homology approach is that only conserved VFs can be identified, while evolutionary distant virulence genes cannot be detected. Often virulence genes can be found on distinct genetic elements in the bacterial chromosome, known as pathogenicity islands (PAI) [69, 70]. Interestingly, genes on PAI usually differ in their nucleotide composition and codon usage bias from genes on the rest of the chromosome. Together with their association with mobile genetic elements, tRNA genes and an accumulation of CRISPR sequences [70] and phage related sequences, PAIs are suggested to be acquired by horizontal transfer [71]. A large collection of PAIs and PAI candidates is stored in the Pathogenicity Island Database (PAIDB) [72]. Most bioinformatics tools developed for the prediction of PAIs rely on composition based methods that employ the specific properties of genomic islands, while some compare closely related genomes. It was shown that combining more than one feature of genomic islands for prediction purposes produces more reliable results [73], for which the application of machine learning methods proved to be useful [74]. A very comprehensive study that compared many GI prediction tools for their user friendliness, methodology, accuracy and precision showed that IslandViewer 4 and GIHunter showed the highest accuracy and precision [75]. Currently only some tools can be applied on draft genomes, which might be overcome by the formation of a reference guided pseudo-chromosome formation that can be obtained by concatenation of sorted contigs [75]. Assembly of PAIs from short reads remains a challenge, for the reason that PAI typically contain repetitive genetic elements such as insertion sequences, which cause the assembly process to generate contig borders at these positions. Additionally these tools might fail, when the sequence composition of the investigated species is similar to the organism from which the genomic islands originated or due to normal variation in sequence composition and occurrence of features typical for PAIs in the genome.

Several machine learning approaches to predict novel VFs have been developed. For example, MP3 [76] uses support vector machines (SVM) and Hidden Markov Model (HMM) to identify virulence protein candidates in metagenomic datasets, even for amino acid fragments typically resulting from the translation of short read sequencing data. The application of a strategy, combining sequence similarity and machine learning, was found to deliver best results for VF prediction [77], an approach that is applied by VirulentPred [78]. VirulentPred applies a two stage cascade SVM learning approach on protein fasta sequences with a background noise reduction step before the classification that can be employed via a web portal (http://203.92.44.117/virulent/index.html). Differently from the previously described sequence based training, some publicly unavailable approaches rely on classification algorithms utilizing sequence associated information from biological repositories such as gene ontology, functional domains and protein-protein network information [79–81]. A recent review concludes that ML-based virulence prediction methods frequently perform worse than BLAST-similarity based approaches [77]. It was shown that the proper definition of an informed, non-random negative dataset is essential and performances commonly fail to generalize in a real-world whole-proteome prediction scenario.

Furthermore other machine learning approaches exist that do not predict VFs as such, but instead predict the pathogenic potential of novel pathogens. Therefore two different concepts exist that have been implemented in different tools: a protein family composition-based [82–84] and a read based classification [85–87]. The first approach depends on the assembly and annotation of a genome and considers only coding sequences, the latter method can be performed on sequencing reads. One advantage of the latter method is that, even when used with few reads predictions are robust, - a useful feature for incompletely sequenced genomes. In any case, the results generated by machine learning approaches should be carefully analysed, given their high dependency on the training datasets and the fact that pathogenicity is not a sufficiently well understood issue [88].

## Typing of the mobilome using WGS data

The chromosome represents the genetic backbone of a bacterium and comprises the majority of information for the development of the organism-specific properties. In addition, bacterial phenotypes can be strongly influenced by the presence or absence of a diverse set of mobile genetic elements (MGEs), which are usually summarized under the term mobilome [89, 90]. MGEs are pivotal for the bacterial adaptation to prevailing environmental conditions and genomic evolution as they force the exchange of genetic information between different bacteria [91]. Variable regions can constitute notifiable amounts of bacterial genomes and are mainly represented by different types of MGEs, i.e. insertion sequences (IS), bacteriophage/phage genomes (prophages), integrative and conjugative elements (ICEs) as well as plasmids [90, 92]. In the pre-WGS era, the determination of the biology and genetics of MGEs was laborious, time-consuming and often limited by the availability of suitable methods. Nowadays, the availability of short- and long read sequencing techniques for WGS determination allows deeper insights into bacterial genomics and provides detailed information of the content and diversity of MGEs (i.e. plasmids, bacteriophages, transposons) [91]. Generally, DNA sequences associated with MGEs of unrelated bacteria can be easily detected as they often exhibit G + C contents that differ to some extents from that of their hosts, indicating earlier events of lateral gene transfer [91]. As MGEs evolve separately from their microbial hosts, they can exhibit a high diversity that might be strongly influenced by the route of their transmission, host bacteria and/or coexistence with other MGEs [91, 93]. Thus, medium- and large-size MGEs often comprise a complex mosaic-like structure exhibiting components (genes, operons, segments) from other elements that might be ancestrally beneficial for the MGEs or its host bacteria. The WGS-based entries in public databases impressively illustrate the extensive diversity of MGEs, which also hamper easy and reliable typing of them [89, 94, 95].

### Plasmid typing

Plasmids are MGEs of high importance as they can contribute to the plasticity of the bacterial genomes by transmitting insertion sequences and transposons that may interact with other prevailing genetic elements (i.e. chromosome, prophages, and other plasmids) [91, 93]. Additionally, these elements can also provoke homologous or non-homologous recombination with the chromosome leading to an exchange of small or large DNA sequences [96]. Plasmids are linear or circular DNA molecules ranging between 1.5 and > 350 kb (megaplasmids) that sometimes integrate into the bacterial chromosome, but often replicate independently as extrachromosomal elements [97]. As they often carry genes that are beneficial for the survival of the host bacteria (i.e. metabolic- & virulence factors, antibiotic and heavy metal resistances, genes for environmental adaptability and persistence) they are important elements for bacterial adaptation [90, 91, 97]. Beside such factors, plasmids can also exhibit genes that are essential for their spread [98]. Traditionally, they were attributed to three different types based on their transmissibility: i) self-transmissible plasmids, also designated as conjugative plasmids, comprise all necessary genetic information to develop a mating pair formation (MPF) complex and DNA transfer replication apparatus, which are required for conjugative transfer; ii) mobilizable plasmids are not self-transmissible and use a MPF complex of another genetic element, while iii), the third type is represented by plasmids that are neither conjugative nor mobilizable [98, 99]. Due to their particular role in exchanging genetic material (horizontal gene transfer), great efforts have been made to develop reliable typing techniques for plasmids. Historically, plasmid-typing was mainly based on incompatibility (Inc) studies of plasmids with other plasmids in the same cell, subsequent restriction profiling and/or DNA-DNA hybridization. The large diversity of plasmid genomes required the development of a reliable and rapid typing system based on DNA-DNA hybridization or PCR amplification of specific replicon DNA units that are essential for autonomously replication (Rep) within a host. The previously described Inc- and Rep-typing procedures both rely on replication factors and provide further insights into the potential impact of the plasmid (i.e. associated with virulence and/or antimicrobial resistance determinants) [100].

There are only some tools for in silico typing of plasmids from WGS data currently available. The manuscript of Orlek and colleagues (2017) provides a comprehensive overview of available tools and strategies for plasmid identification [100] of which only some are addressed below. One of the most popular tools, PlasmidFinder [96], enables the detection of plasmid replicons and assigns the requested plasmids to the respective Inc. group of the previously used Inc./Rep-typing schemes [100]. PlasmidFinder further provides information on the similarity values of the requested sequence to a closely related reference. Users that are interested in a more thorough typing of plasmids can further use the pMLST tool that provides plasmid MLST allele sequence and profile data from public databases for molecular typing (https://pubmlst.org). PlasmidFinder is well established for in silico analysis of plasmids from Enterobacteriaceae and some Gram-positive bacteria, but lacks information on plasmids from a broad range of other bacteria [96]. PLACNETw, another tool for plasmid reconstruction from WGS data, uses information about scaffold links and coverage of the WGS assembly, nucleotide comparison to reference plasmids, and plasmid features (i.e. replication initiator proteins) for in silico prediction. This tool also provides additional features for plasmid visualization and further downstream analysis [101]. Plasmid Profiler is a pipeline that performs comparative plasmid content analysis and provides a heatmap of the plasmid content in WGS data. For plasmid prediction, the pipeline initially identifies plasmids of the reference database that are represented in the reads using the K-mer Analysis Toolkit (KAT) and develops individual isolate plasmid databases. Subsequent analysis is conducted using SRST2 to identify plasmid matches from the individual isolate plasmid databases. Finally, the BLAST suite is used to identify the incompatibility group and specific genes of interest on the plasmid sequences. Thereafter the identified matches are scored on a combined measure of maximized coverage and minimized sequence divergence. The program provides a static and an interactive heatmap as well as a tabular summary of the results. Beside WGS data the user further needs a reference plasmid database and replicon/gene of interest database for comparative analysis [102]. PlasFlow is a scripts-based plasmid sequence prediction tool for metagenomic data that relies on neural network models. The models were trained on full genome and plasmid sequences and are thus able to differentiate between chromosomes and plasmids. Beside this information, the tool also provides thresholds that allow for an assessment of the prediction quality [103].

There are also some tool independent options for the prediction of plasmid-based sequence contigs in WGS data [100]. The first prediction option is based on the copy number of the plasmids. Usually, small- and medium-size plasmids provide a higher copy number per bacteria than the chromosome [104]. Thus sequence contigs that are based on small or medium-sized plasmid usually yield higher sequence coverages than chromosomal contigs. Given that large plasmids often exhibit similar copy numbers as the chromosome this option might be only suitable for reliable prediction of small and medium-sized plasmids. The second option for plasmid prediction is based on the predominantly circular structure of plasmid molecules. Thus, DNA contigs exhibiting terminal redundant sequences might represent plasmid contigs. However, a lot of DNA molecules, especially transposons and insertion sequences also provide DNA fragments with terminal repeats leading to false-positive plasmid predictions without further analysis.

### Phage typing

The content and composition of prophages in bacteria is of particular importance for genome diversification, as the repertoire of bacteriophage (phage) sequences can represent a notifiable amount of the variable gene content among different bacterial isolates. The great majority of the frequently sequenced bacteria are lysogens and therefore represent a huge source of prophages [105, 106]. Prophages are genomes of temperate phages that have infected a susceptible host bacterium, were they either integrate into the chromosome or exist as circular or linear plasmids. During the lysogenic lifestyle, prophages coexist with their hosts in a latent form without producing virus particles. Specific cellular stress signals (i.e. temperature, antibiotics, UV radiation) can activate the lytic lifestyle, in which virus propagation is initiated and cellular lysis occurs. As the genomes of temperate phages usually exhibit additional non-essential genetic information, prophages often provide genes that potentially encode beneficial components for the host (i.e. gene products involved in a number of bacterial cellular processes, antibiotic resistance, stress response, and virulence) [105, 106]. For most of the temperate phages functional information on their accessory genome is widely unknown, as only some of the identified genes encode products of predictable functions. Furthermore, classification of bacterial viruses is often challenging as bacteriophages belong to the most common and heterogeneous entities of the biosphere. It has been estimated that more bacteriophages (> 10^31^) appear on the earth than bacteria (> 10^29^) [107]. In the past, phages were mainly classified on the basis of the morphology of their virion particles as well as their DNA structure. Nowadays, the genetic structure and organization of their genomes are also pivotal for their classification [108].

For the prediction of prophage sequences within WGS and metagenomics data from bacterial genomes, several tools have been developed. A comprehensive summary on available tools and their properties was recently published by Song et al., 2019 [109]. Most of the currently available programs (i.e. Prophage Hunter, MARVEL, PHAST or PHASTER, MetaPhinder, VirSorter, PhiSpy) use similarity matching with entries of the phage/prophage/virus databases and are based on specific phage genome features (i.e. components for lysis, integration, replication, lifestyle regulation, DNA packaging, virion assembly). Some of them, e.g. Prophage Hunter, further use machine learning classifier to assess the status of the prophages. For some of the tools additional functions are available (i.e. annotation of gene products or the prediction of the attachment site), which might be advantageous for the assessment of the predicted prophage sequences. Specifically the prediction whether a prophage might still be active or only represents a remnant DNA artefact (cryptic prophage that was inactivated due to bacterial defense systems or mutational decay) is important in order to assess the impact and its potential for further spreading [105, 106]. Overall, many of the tools provide a good performance in detecting prophage sequences in bacterial WGS or metagenomics (i.e. MARVEL) datasets and can often be used by researchers without programming skills (i.e. Prophage Hunter, PHAST/PHASTER, VirSorter). However, in silico assessment of prophages might still be challenging, especially if bacterial WGS data of underrepresented organisms is analysed and the used phage/prophage/virus databases lack data on their bacterial viruses [109]. Due to the huge number of prophages and their high diversity further efforts are needed for reliable prophage prediction and activity assessment as the identification of active prophages is crucial for studying co-evolution of phage and bacteria [105, 106].

### Transposable elements

Transposable elements are integral parts of bacteria and consist of insertion sequences and transposons. While insertion sequences are simply structured, short DNA elements (< 5 kb with usually 1–2 coding sequences) only comprising genes that facilitate their transmission, transposons are larger (> 5 kb) and highly variable in their gene content. Beside genes for movement, transposons are more complex versions of insertion elements that further encode additional genetic information (i.e. metal and antibiotic resistance determinants) that might be beneficial for the survival or the adaptation of the bacteria. Usually, transposable elements exhibit highly variable frequencies of transposition ranging between 10 and 7 to 10–2 per generation. For movement, the DNA of the target sequence and of the ends of the transposon is cut. Thereafter, the ends of the transposon and target DNA are joined and replication takes place either by a replicative or non-replicative mechanism, in which the complete transposon or only short fragments at the end of the insertion site are replicated, respectively. Insertion elements usually exhibit short terminal inverted repeats at both ends, which provide target sites for homologous recombination. IS elements can cause rearrangement or deletion and contribute to the plasticity of the genome, bacterial adaptation and genome evolution.

A diverse set of tools for IS and/or transposon prediction is available. The publication of Bergman and Quesneville [110] provides a good overview on available tools and their prediction strategies. A comprehensive actively curated summary of IS prediction tools is also available on the homepage of the Bergman laboratory (http://bergmanlab.genetics.uga.edu/). In general, prediction tools for transposable elements follow a broad range of approaches that can be based on de novo repeat detection, sequence homologies, the genetic structure and/or comparative analysis. Tools (i.e. Reputer, RepeatMatch, RepeatFinder, PILER, ReAS) using de novo repeat detection are typically used for the identification of novel transposable elements. This approach relies on the identification of DNA repetitions in assembled data and is therefore dependent on sequence quality and the used assembling algorithm. Nevertheless, differentiation between repeats from transposable elements and other repetitive sequences is still a challenge. Tools that are based on the homology-matching approach for the detection of similarities to coding sequences of known transposable elements are thus biased and dependent on the current level of knowledge. Furthermore, these tools also fail to identify transposable elements without coding sequences. Tools predicting transposable elements on the basis of the genetic structure (i.e. LTR_STRUC, SMaRTFinder) rely on identification of repeat regions. The approach has been mostly used for the prediction of long terminal repeat retrotransposons. Other approaches rely on comparative genomic-based methods [111], that search for large insertions in multiple alignments that were created by transpositions. However, methods using this approach are dependent on the activity of the transposable elements. Therefore, without any transposition (i.e. if ancestral transposable elements are present) the tools will not detect transposable elements. As all of these approaches rely on important features of transposable elements, best practice will be observed with tools implementing more than one of them [110].

## Typing of antimicrobial resistance

Naturally, antimicrobials are produced as secondary metabolites by bacteria and fungi from soil and marine habitats to inhibit the growth of other organisms and thus to gain a competitive advantage [112]. When cells are able to grow in presence of an antibiotic, they are classified as antimicrobial resistant. Antimicrobial Resistance (AMR) is a natural phenomenon, as old as the antibiotic substances themselves and many bacteria co-existing with antimicrobial-producers have developed intrinsic resistant mechanisms [113]. In addition, AMR can also be acquired by formerly susceptible bacteria. History has shown that shortly after the introduction of a certain antimicrobial in human or veterinary medicine, resistant bacterial clones emerged and spread in human and animal populations. This phenomenon was attributed to the selection pressure caused by antimicrobial usage [114]. Development of AMR in human pathogens is accompanied by increasing mortality rates and economic costs and represents a major public health burden in the twenty-first century [115]. Generally, AMR can occur through various mechanisms including: i) degradation or enzymatic modification of the antimicrobial, ii) overproduction, protection or modification of the antimicrobial target, iii) antimicrobial efflux and iv) change in cell permeability resulting in restricted access to the target site [116–118]. Formerly susceptible microorganisms can acquire AMR either by chromosomal point mutations, through overexpression or duplication of antimicrobial target genes, or through acquisition of antibiotic resistance determinants by horizontal gene transfer [118, 119].

To measure AMR in bacterial isolates conventional phenotypic screening can be performed to determine the concentration of a certain antimicrobial necessary to prevent bacterial growth (minimum inhibitory concentration (MIC) measurement) [120]. Commercial and standardized 96-well broth microdilution panels belong to the most widely used methods to test bacterial growth in different antibiotics and antibiotic concentrations [121]. The determined MIC values are compared to clinical breakpoints or epidemiological cut-off values to decide whether a bacterial isolate is susceptible or resistant to a certain antibiotic [120].

To closely investigate the mechanism underlying AMR, a genotypic characterization of isolates is necessary. Nowadays, AMR genes and point mutations associated with AMR can be identified in WGS data [120]. When working with short-read sequencing data, AMR genes can be detected either using assembly-based or read-based approaches [118]. In the assembly-based approach, short-read sequencing reads are first assembled into contigs and AMR genes are identified using BLASTN-based tools comparing the derived draft genomes to AMR reference gene databases [118, 120, 121]. Examples for assembly-based approaches include the ResFinder tool (now including PointFinder) searching the ResFinder database and the Resistance Gene Identifier (RGI) searching the Comprehensive Antibiotic Resistance Database (CARD) [118, 122, 123]. Both tools are able to identify acquired resistance genes as well as point mutations and are available as web-based or standalone versions [118]. In read-based approaches, short-reads are either aligned to reference databases using pairwise alignment tools, as implemented by SRST2, or split into shorter k-mers which are subsequently mapped to a k-mer database obtained from reference sequences, as implemented in KmerResistance or the latest ResFinder 3.2 version (when submitting raw reads) [64, 118, 124]. These methods have in common that they can detect acquired antimicrobial resistance genes, but are not able to identify point mutations associated with antimicrobial resistance. Moreover, information about regulatory elements located upstream or downstream of resistance genes are not provided when using read-based approaches [118]. Although these methods are less computationally demanding as assemblies are not required, they provide an advantage when dealing with metagenomics samples, as resistance genes in less abundant organisms from complex samples can be identified despite low coverage [118]. For reliable resistance gene identification, resistance gene databases have to be continuously updated. One disadvantage of common AMR databases is, that novel or remote homologous AMR genes from less well studied bacteria might be missed, for the reason that these databases are heavily biased towards easy-to-cultivate human pathogens [118]. One approach to overcome this bias is, to use databases which include antibiotic resistance determinants from metagenomics samples, e.g. ResFinderFG [125]. Another approach is to use Hidden Markov model-based databases such as Resfams, which were developed to identify potential AMR genes with the same function, but low sequence identity to known AMR genes [118, 126].

To predict the resistance phenotype (MIC values) from genotypic data, rules-based or machine learning approaches might be used [127, 128]. Rules-based algorithms predict AMR phenotypes using curated reference sets of genes and point mutations involved in resistance, whereas machine-learning algorithms use a model built from a training set comprised of WGS and phenotypic data of resistant isolates [127, 128]. Rules-based methods can be used, when the factors contributing to AMR are well known. When information about the underlying mechanism of resistance is insufficient, prediction of MIC values based on reference-free machine learning may be the better approach. Nguyen et al. [127] developed extreme gradient boosting (XGBoost)-based machine learning models for the prediction of MICs for 15 antibiotics in non-typhoidal *Salmonella* strains from whole-genome sequencing data. Nguyen and colleagues used datasets with available WGS and phenotypic AMR data to train their models, which were subsequently able to predict MICs of other *Salmonella* strains without information about the resistance phenotype or genes involved in molecular resistance mechanisms. This reference-free approach for predicting MIC from whole-genome sequencing data can be applied to other pathogens relevant for surveillance or clinical diagnostics and might even be used to detect new genomic features involved in AMR [127]. However, complete replacement of phenotypic AMR measurement by molecular AMR prediction approaches is not advised, given that bacterial strains continue to evolve and new resistance mechanisms are going to emerge, which may be overlooked as they are not represented in AMR databases or in the datasets used to train machine learning models. Therefore, phenotypic testing of a representative genomic diversity of strains needs to be maintained to ensure that genotypic AMR results do not diverge from the true AMR phenotype over time [129].

## Serotyping prediction

Subtypes within different genus of food-born pathogenic bacteria can be differentiated by their highly variable antigenic surface structures. The presence of an antigen can be detected through a series of immunological tests, in which cells are mixed with specific antisera to induce agglutination. Derived from these serological tests subtypes are commonly known as serovars or serotypes. The distinction of foodborne bacteria into serovars, starting from the 1930s has proven extremely useful for the reason that characteristics such as host specificity, virulence and pathogenicity usually correlate well with serovar assignments. Consequently, serovar assignment has provided scientists, public health experts and the general public with an effective terminology and a perquisite for monitoring and surveillance schemes. To date, about 2600 different *Salmonella* serovars have been identified [130]. Within *Escherichia coli* there are approximately 190 known serovars [131], while *Shigella* spp. are differentiated in 54 serovars [132]. There are 47 recognized serovars of *Campylobacter jejuni* [133] and 13 serovars for *Listeria monocytogenes* [134]. In general, serotyping is based on the somatic O antigen, a cell surface protein and the H antigen, which forms part of the flagella (for serotyping of *Shigella* only the O antigen is of consideration). Serotyping of *C. jejuni* is slightly different and is based on the capsule polysaccharide (CPS) [133]. Each known antigen is assigned a number and letter code, which are then combined into a seroformula according to an established scheme, such as the White-Kauffmann-Le Minor scheme for *Salmonella* [9], the Shigatoxin-producing *E. coli* (STEC) scheme [135] and the Penner scheme for *C. jejuni* [136].

Although traditional laboratory serotyping does not require expensive equipment, it is time- and resource consuming, as well as labour-intensive and can be limited by the non-expression of surface antigens. To overcome these drawbacks, several in silico methods have been developed in recent years, which analyse sequencing data derived from WGS to predict the serovar of an isolate. An overview of currently available tools for in silico serovar prediction is shown in Table 3.

**Table 3:** List of different tools for in silico serovar prediction

Tool (Reference)	Species	Method	Source
SeqSero [137]	*Salmonella* spp.	mapping of raw reads to database of O and H antigen alleles	command line: https://github.com/denglab/SeqSero
			Web tool: http://denglab.info/SeqSero
SeqSero2 [138]	*Salmonella* spp.	similar SeqSero, additional performs rapid serotype prediction based on unique k-mers of serotype determinants	command line: https://github.com/denglab/SeqSero2
Salmonella TypeFinder	*Salmonella* spp.	in addition to running SeqSero, determines 7 gene MLST and infers serovar from sequence type according to Enterobase database	Web tool: https://cge.cbs.dtu.dk/services/SalmonellaTypeFinder/
SISTR [139]	*Salmonella* spp.	mapping of database of O and H antigen alleles against genome assembly, additionally, determines cgMLST and infers serovar from cgMLST clustering result	command line: https://github.com/phac-nml/SISTR_cmd
			Web tool: https://lfz.corefacility.ca/sistr-app/
MOST [140]	*Salmonella* spp.	determines 7-gene MLST and reports number of respective serovar – ST matches from the PHE/Achtmann database	command line: https://github.com/phe-bioinformatics/MOST
Serotype Finder [141]	*E. coli*	mapping of raw reads to database of O and H antigen alleles, or mapping of antigen alleles against genome assembly	command line: https://github.com/Papos92/ecoli_serotyper
			Web tool: https://cge.cbs.dtu.dk/services/SerotypeFinder/
ECTyper	*E. coli*	Information not available	command line: https://github.com/phac-nml/ecoli_serotyping
EBEis from the Enterobase Tool Kit	*E. coli Shigella* spp.	mapping of database of O and H antigen alleles against genome assembly	command line: https://github.com/zheminzhou/EToKi#ebeis%2D%2D-in-silico-serotype-prediction-for-escherichia-coli%2D%2Dshigella-spp
Seq_typing	*E. coli*	mapping of raw reads to database of O and H antigen alleles, or mapping of antigen alleles against genome assembly	command line: https://github.com/B-UMMI/seq_typing
LisSero [134]	*Listeria monocytogenes*	mapping of database of 5 DNA regions (*lmo1118, lmo0737, ORF2110, ORF2819, prs*) against genome assembly	command line: https://github.com/MDU-PHL/LisSero

Different strategies can be applied to infer serovar predictions from sequencing data. The most common is the detection of sequence differences that cause variations in either the O or the H antigen. In general, tools that follow this approach, such as SeqSero [137] and SerotypeFinder [141], implement a mapping alignment, which aligns the obtained sequencing reads to a reference database of antigen allele sequences and then assign the antigenic formula and the serovar name based on the best scoring alignments. It is also possible to break reads into k-mers, which are then compared to the frequency of unique k-mers of serotype determinants as implemented in SeqSero2 [138]. A difficulty of these approaches is that usually there is no single gene encoding the antigens. For example the O antigen of *Salmonella* is determined by the *wzx* flippase gene the *wzy* polymerase gene as well as additional genes from the *rfb* cluster. Another issue is that some closely related serovars share the same antigenic seroformula, but feature minor differences in their O antigenic factors, such as *S. enterica* serovar Kottbus and *S. enterica* serovar Ferruch.

Another approach for in silico serovar prediction is to infer serovars from multi-locus sequence types, e.g. the *Salmonella* 7-gene Multi-Locus Sequence Typing (MLST) scheme [9], as implemented in MOST [140]. Sequence types have been shown to correlate well with serovars, although one weakness of this approach is that sometimes more than one serovar is associated with a sequence type. Furthermore serovar prediction fails when an isolate features a novel sequence type, for which no associated serovar is available in the database. A continuation of this strategy is the determination of serovar predictions from cgMLST, as implemented in SISTR [139]. In this method the cgMLST of an isolate is determined and a pairwise distance matrix between any two genomes is computed. From the distance matrix, isolates are hierarchically clustered and the serovar is predicted based on the dominant serovar of the respective cluster. This whole genome based method refines serovar predictions by considering the phylogenetic context and is especially useful when draft genome assemblies contain incomplete antigenic regions.

In addition to these methods, several studies have further investigated the utility of lineage-specific gene markers for the identification of polyphyletic serovars [142–144]. However, we are not aware of any currently publicly available program that implements the findings from these studies. Furthermore, a recently published package for R explores the possibility to predict serovars of *Salmonella enterica* based on the sequence of CRISPR spacer pairs [145].

Benchmarking studies and comparative performance assessment of in silico serotyping tools attest a medium to high correlation with conventional serotyping (70–95% agreement) [146–148], which is likely to improve further in the future. It is important to note that all tools, regardless of their respective approach rely heavily on the underlying databases. Most tools do not update reference databases, rendering prediction results less accurate for novel and / or rare serovars. Furthermore the quality of the sequencing data can have an impact on robust prediction, especially if tools require assembled draft genomes as input. Since there is great variety in assembly algorithms, the chosen algorithm may also have an effect on serovar predictions [147].

The availability of online web interfaces for different tools (for example SISTR, SeqSero, SalmonellaTypeFinder, SerotypeFinder), make in silico serotyping tools easily and widely accessible. Despite their advantages they are not suitable for high-throughput, independent, reliable and reproducible results generation. Only their command-line program versions can be integrated into in-house bacterial characterization analysis pipelines, which allow rapid, efficient, customized and controlled bioinformatics analysis of WGS data on a day-to-day basis.

Overall, in silico serotyping is a rapid, efficient, cheap and reproducible analysis process. However, further benchmarking and comparison studies are needed to reliable evaluate the available tools. Furthermore, continuously updated curated and extensive databases, as well as standardization of serovar names are needed for accurate and comparable in silico serovar prediction.

## WGS analysis platforms

As discussed previously, a great variety of methods and tools is available to analyse and characterize bacterial pathogens. Many of these tools are implemented for Unix environments and require at least some bioinformatics expertise for usage. To enable epidemiologists, microbiologists and other researchers to interpret the biological coherencies, there is a variety of online platforms including commercial software available for collection, analysis and visualization of sequencing data [149, 150]. These platforms generally start their analyses from raw sequencing data or assemblies and rely on different approaches for organization of metadata, sequencing data, and various analysis steps. The major distinction of all presented platforms are, whether they use a SNP or an allele calling (gene-by-gene) approach for hierarchical clustering to calculate phylogenies from WGS data (compare Table 4). Most platforms implementing cgMLST provide their own cgMLST schemes or host a collection of existing ones. While the choice of scheme is vital for the comparability of results, the number of well tested schemes for non-model organisms is limited. A list of currently available schemes is given in Table 2. If no suitable scheme is available, users can generate their own scheme, by using tools such as Ridom SeqSphere+ [157] or chewBBACA [158], always provided that a sufficient number of reference genomes is available.

**Table 4:** Key characteristics of selected platforms

Platform	Reference	Typing approach	Central instance/ Local instance	Commercial (C)/ Academic (A)
BIGSdb	[151]	wg/cgMLST	Both possible	A
Bionumerics	http://www.applied-maths.com/applications/wgmlst	wg/cgMLST and SNP	Both possible	C
CGE	[152]	cgMLST and SNP	Cloud	A
COMPARE	[153]	cgMLST and SNP	Cloud	A
Enterobase	[16]	wg/cgMLST and SNP	Cloud	A
INNUENDO	[154]	wg/cgMLST	Local	A
IRIDA	[155]	wg/cgMLST and SNP	Local	A
NCBI Pathogens	[156]	wg/cgMLST and SNP	Cloud	A
PathogenWatch	https://pathogen.watch	cgMLST	Cloud	A
PATRIC	[68, 150]	SNP	Cloud	A
SeqSphere+	https://www.ridom.de/seqsphere/	wg/cgMLST	Local	C

Platforms can also be differentiated by whether they are web-based or run in local instances. While web-based tools are often free for use and do not require computational power from the user, they often demand users to deposit the analyzed data in public repositories. This is especially challenging for hospital laboratories and private sector companies, who are often hesitant to share their data publically. However, it is a necessity to keep databases up to date in order to be able to detect potential links between isolates from different sources [159].

All platforms have their own unique set of pipelines and tools for the analysis of WGS of different bacterial species. Fundamental questions for many real-world scenarios include analyses such as AMR detection, pathotyping and virulence gene detection, serotyping and phylogenomics. Each of these features is presented for the selected tools in Fig. 1. Table 4 provides an overview of the most widely used platforms and their specifications with regard to the functionality described previously. A more detailed overview of some of these tools has been composed in an EFSA/ECDC technical report [149].

The major advantage of applying online platforms or commercial software tools for WGS analyses is that usage requires no or only limited bioinformatics knowledge. Since users often have no insight regarding the underlying algorithms and parameters of the tools, this might lead to unreliable analyses and in last consequence to misinterpretation of the result data. Therefore, training of users and well-written documentation of platforms and tools is a vital prerequisite for effective usage of these platforms.

## Future directions

Many typing tools and databases have been developed to allow the meaningful analyses of WGS data for a variety of investigations. Sequencing technologies are still rapidly evolving, generating more accurate data, for less money with greater user-friendliness. This leads to the technology being implemented on a broad, worldwide scale. The current dynamic in the development of new techniques and analysis tools and the transformation of these into routine disease surveillance, will require a great amount of standardization to ensure the comparability of WGS data and results between laboratories. One major issue is the harmonized assignment of new sequence types according to cgMLST/wgMLST, which theoretically would require a large centrally organized curated database. One workaround solution could be the implementation of allele hashing instead of the use of simple allele numbers, since hash-tagging allows for the decentralized allocation of sequencing types. Considering the great variety of typing tools, as well as their continuous development, standardization may not be a viable option. Instead, the careful validation of those tools with well-documented data test sets could ensure that the results are “truth”. By this approach, WGS data of bacterial isolates might not be directly comparable, but interpretation of result data and derived conclusions would be overall similar. Standards should be developed for the internationally accepted validation of typing tools [160] and benchmarking data sets for validation shall be extended. This would make the need for a specific validated cgMLST nomenclature system for a particular bacterial species obsolete. The databases underlying bioinformatics tools, e.g. for serotyping or virulence typing, need to be professionally curated to avoid erroneous results. This demands human and hardware resources and needs to be addressed to decision makers on a global scale e.g. FAO, WHO, or OECD. International biological repository institutions for sequences such as ENA (Europe), NCBI (U.S.A.) and DDBJ (Japan) would be well suited to host such tools. The NCBI Pathogen Detection Pipeline [161] is a promising development for a standardized analysis pipeline, especially if shared with a broader scientific community and which could be expanded to include a variety of tools for analyzing WGS data (e.g. cgMLST, serotyping, virulence).

SNP-based mapping approaches are problematic for the comparisons of genetically highly diverse bacteria, such as *Campylobacter* spp. and *Helicobacter pylori* due to large scale fluctuations disrupting the clonality of the species. For those pathogens, typing approaches could be more effective in describing the evolutionary relationships between these diverse microorganisms. Although reference-free assembly followed by gene-by-gene approaches are more robust for horizontal gene transfer events misinterpretation is still possible. Better visualization tools for the examination of the phylogenetic, geospatial and temporal distribution of isolates on a global as well local scale are urgently needed. The visualization of phylogenomic data in combination with metadata is a crucial step in understanding the complex relationships between isolates, informing further actions and decisions. A plain data collection in regard to surveillance of pathogens is not sufficient. Some projects such as Microreact (https://microreact.org) or NextStrain (https://nextstrain.org/) have developed tools for this purpose, but these need to be more broadly accessible and applicable for official laboratories involved in routine surveillance. We believe that visualization of typing results could be much improved, leading to a deepened understanding of the evolution of pathogens and disease outbreaks.

Beside good visualizations, successful interpretation of typing data requires equal input and expertise from molecular biologists, epidemiologists and bioinformaticians. The importance of all three fields should be reflected in team structures, education and research programs. In future, most phenotypical and PCR based methods can be substituted with in silico WGS analyses. Others, such as traditional phenotypic antimicrobial resistance assays will continue to be of high relevance since there is still an insufficient understanding of the physiological links between geno- and phenotype. The decision which types of analysis can be switched from traditional microbial testing to WGS will heavily depend on evaluation and validation studies, as well as on a general increase of knowledge and understanding of WGS data analysis within the community. Scientists who analyse WGS data currently use software which are built on mechanistic model-based approaches for comparative genomics and genome characterization. Recently however, bioinformaticians have taken advantage of artificial intelligence and its sub-discipline machine learning [162]. Whilst mechanistic model-based systems are based on simplified mathematical formulations considering input-output relationships, machine learning makes predictions on large-scale datasets that bypass the need for causality [163]. In the future, typing approaches could tremendously benefit from this trend, with the potential to refine these methods with an unprecedented resolution [164].

## Conclusions

Whole genome sequencing technologies have pushed the development of advanced typing approaches for bacterial genome comparisons, which are primarily based on SNP and gene-by-gene analyses. Both methods provide often similar conclusions, but may vary in their resolution and suitability for different species and epidemiological cases. The construction and interpretation of phylogenetic trees derived from these data, makes it possible to identify transmission events and to understand the dynamic of outbreaks, which is still a challenge. As more data will be generated and as more documented examples of genetic relationships in terms of spatial and temporal variations will be described, the better we will understand the evolution of bacterial species and their variants in human, animal, food and the environment. The high resolution of WGS nullifies simple thresholds of relatedness as applied for classical molecular typing methods. We believe that the public and animal health, food safety and environmental scientific disciplines should extend their collaboration to benefit from this immense opportunity to build more efficient One Health tools and databases. Furthermore new approaches such as machine learning for robust phylotyping and for the interpretation of WGS data need to be explored and implemented where their usefulness is demonstrated. The development of advanced open-source and easy-to-use typing tools will play a central role in achieving this goal. However, a successful routine global surveillance requires the consolidation of the developed tools as a perquisite for the setting of international standards.

## Data Availability

There is no Supplementary Material for this article available.

## Abbreviations

AMR: Antimicrobial resistance
BLAST: Basic local alignment search tool
cgMLST: Core genome multilocus sequence typing
DNA: Deoxyribonucleic acid
GUI: Graphical user interface
HierCC: Hierarchical clustering of cgMLST
HMM: Hidden markov model
ICE: Integrative and conjugative element
IS: Insertion sequences
MGE: Mobile genetic element
MIC: Minimum inhibitory concentration
ML: Maximum likelihood
MLEE: Multilocus enzyme electrophoresis
MLST: Multilocus sequence typing
MLVA: Multilocus variable-number tandem-repeat analysis
MPF: Mating pair formation
MS: Minimum spanning
NGS: Next-generation sequencing
NJ: Neighbor-joining
PAI: Pathogenicity island
PCR: Polymerase chain reaction
PFGE: Pulsed field gel electrophoresis
RAxML: Randomized axelerated maximum likelihood
SNP: Single-nucleotide polymorphism
ST: Sequence type
SVM: Support vector machine
VF: Virulence factor
wgMLST: Whole-genome MLST
WGS: Whole genome sequencing
